# Effect of Long-Term Supplementation of AZOMITE (Hydrated Sodium Calcium Aluminosilicate) in Finishing Diets on Growth Performance, Dietary Energy, and Carcass Yield of Hairy Lambs

**DOI:** 10.3390/ani14203018

**Published:** 2024-10-18

**Authors:** Claudia A. Vizcarra-Chávez, Jesús D. Urías-Estrada, Elizama Ponce-Barraza, Alfredo Estrada-Angulo, Yesica J. Arteaga-Wences, Beatriz I. Castro-Pérez, Jorge L. Ramos-Méndez, Luis Corona, Armando Gomez-Vázquez, Alejandro Plascencia

**Affiliations:** 1 Faculty of Veterinary Medicine and Zootechnics, Autonomous University of Sinaloa, Culiacan 80260, Mexico; claudia.vizcarra2208@gmail.com (C.A.V.-C.); david.urias@uas.edu.mx (J.D.U.-E.); elizama.ponce@hotmail.com (E.P.-B.); alfred_vet@hotmail.com (A.E.-A.); arteaga.yesi.92@hotmail.com (Y.J.A.-W.); laisa_29@hotmail.com (B.I.C.-P.); ramos.jorge.92@outlook.com (J.L.R.-M.); 2 Faculty of Veterinary Medicine and Zootechnics, National Autonomous University of Mexico, Mexico City 04510, Mexico; gochi@unam.mx; 3 División Académica de Ciencias Agropecuarias, Universidad Juárez Autónoma de Tabasco, Villahermosa 82680, Mexico; armando.gomez@ujat.mx

**Keywords:** feedlot lambs, volcanic clay, AZOMITE, productivity, dietary energy, carcass

## Abstract

**Simple Summary:**

The use of volcanic clays as a feed additive in ruminant diets has been gaining greater interest in recent years. Due to their mineral profile and physicochemical nature, they have present characteristics that can promote health and performance improvements. However, there exists a controversy around the results observed with clays as feed additive on feed efficiency in ruminants. It has been argued that the magnitude of the response to clay supplementation can be mediated by several factors, among them the concentration and mineral profile. AZOMITE is a volcanic clay which has been reported to contain over 70 measurable minerals, including Fe, Mg, Mn, Se, Zn, Cu, and rare earth elements (REEs) such as lanthanides, in such a way that it contains up to five times more mineral elements than other clays commonly used as feed additives. In this study, it can be determined that AZOMITE clay can be included up to 1.5% in finishing diets for lambs without negative effects on the intake of water and feed; an inclusion of 0.75% in the diet showed greater improvements in feed efficiency and dietary energy utilization, whereas an inclusion of 1.50% showed the greater improvements to hot carcass weight and dressing percentage without effects on visceral organ mass.

**Abstract:**

The aim of this study was to evaluate growth performance, dietary energy utilization, and carcass characteristics (carcass weight, dressing percentage, and visceral organ mass) of feedlot lambs fed different levels of AZOMITE (AZO), a source of volcanic clay composed of hydrated sodium calcium aluminosilicate, the same as that included in a finishing diet. For this reason, 36 Pelibuey × Katahdin crossbred intact male lambs (18.81 ± 3.04 kg initial weight) were used in a feeding trial lasting 81 d. Lambs were grouped by initial weight and assigned within six weight groupings to 18 pens in a randomized complete block design. Diets, offered ad libitum, were corn-based finishing diets with an 88:12 concentrate-to-forage ratio supplemented as follows: 1) no AZO inclusion (CTRL), 2) AZO inclusion at a 0.75% level (0.75AZO) in diet dry matter, 3) AZO inclusion at a 1.50% level (1.5AZO) in diet dry matter. Cracked corn was replaced by AZO. After the feeding trial was finished, lambs were slaughtered and carcass weight was registered and dressing percentage was calculated. The data were analyzed as a randomized complete block design, with the pen as the experimental unit. Water consumption and dry matter intake were not affected (*p* ≥ 0.11) by supplemental AZO. The incorporation of AZO into the diet increased gain efficiency and observed dietary net energy (NE), being maximal with 0.75% AZO inclusion (quadratic effect, *p* = 0.02). The observed-to-expected dietary NE in the control group was as anticipated (1.00) according to the estimated energy based on the ingredient composition in diet, while in the 0.75AZO group, the observed dietary energy was 6% above expected, indicating a greater efficiency in energy utilization destined to growth (quadratic effect, *p* = 0.006). Lambs that were fed the AZO treatment showed an improvement (linear effect, *p* = 0.04) in hot carcass weight (HCW), and tended (linear effect, *p* = 0.06) to improve dressing percentage (DP) as AZO was increased in the diet. Visceral organ mass was not affected by the treatments. It is concluded that AZOMITE clay can be included up to 1.5% in finishing diets for lambs without negative effects on the intake of water and feed. Lambs that received diets supplemented with 0.75% AZO showed greater improvements in feed efficiency and dietary energy utilization, but an inclusion of 1.50% resulted in greater improvements to HCW and DP. This is the first report regarding the effects of AZO supplementation in the dietary energy utilization of fattening hairy lambs. Further research about the effects of AZO supplementation on ruminal and total tract digestion, carcass and meat quality, and the health of lambs is needed in order to more deeply understand the effects of AZO on its productivity performance.

## 1. Introduction

The use of volcanic clays as a feed additive in animal diets has been gaining greater interest in recent years [1]. “Volcanic clays” refers to the rock generated from volcanic parent materials in areas that have experienced volcanic or tectonic activity. The clays themselves are just degraded lava rock. Among the most used clays are those from the aluminosilicate family in which zeolite, kaolin, and bentonite, among others, are found. As its name indicates, the major components are silica (SiO_2_) and alumina (Al_2_O_3_), but they can fix calcium hydroxide when mixed with lime or an alkaline product and water is present, thus forming hydrated sodium calcium aluminosilicate (HSCAS) [2]. In this manner, although volcanic clays have a certain similarity in mineral composition, depending on the region where they are formed, there are particularities regarding the presence and/or concentration of certain minerals that provide special physical and chemical characteristics [3]. A volcanic clay mined in Utah, United States of America, was formed from ashes which settled into an ancient seabed which was fed a combination by many rivers rich in minerals and rare elements [a dacitic (rhyolitic) tuff breccia]; as a result, this volcanic ash created the AZOMITE mineral composition unique to its deposit. The deposit has been reported to contain over 70 measurable minerals, including Fe, Mg, Mn, Se, Zn, Cu, and rare earth elements (REEs) such as lanthanides, in such a way that it contains up to five times more mineral elements than other members of the aluminosilicate family (i.e., zeolites) commonly used in animal feed [4]. The greater content in the mineral profile and REEs for AZO could represent an advantage for animal productivity when it is supplemented in adequate levels. In this sense, inclusions of 0.25 to 1.0% of AZO in diets for broilers have improved weight gain and/or feed efficiency [5]. The increases in growth rate and feed efficiency in the broilers were explained by increases in nutrient digestibility and by improvements in mineral retention [6]. Moreover, AZO included 0.25% increased N retained in broilers optimizing the CP utilization from the diet [7]. In a study performed by Teng et al. [8], they observed that antioxidant response and intestinal permeability were improved, increasing growth performance and feed efficiency in broilers during the first 26 d of age. Similar positive responses have been obtained when AZO was supplemented in diets for fishes [9]. The positive effects observed in birds and fish (energy, protein, and mineral retention improvements and antioxidant effects) are the effects sought with the inclusion of feed additives. Surprisingly, information on the impact of the use of AZO in finishing diets for ruminants was not available at the time of preparation of this manuscript. Regarding small ruminants, recent studies have shown that the mineral requirements of finishing hairy lambs are greater than those indicated in current standards [10,11]. Thus, due to the profile and concentration of minerals contained in AZO (macro, trace, and rare earth elements), it could be a complementary source of diet minerals to hairy lambs. Due to the above revelation, we hypothesized that AZO could have positive effects on the rate of weight gain and dietary energy utilization which could positively affect the carcass dressing percentage of fattening lambs. For this reason, the objective of this study was to evaluate the effects of AZO inclusion in finishing diets on growth performance and dietary energy utilization in feedlot hairy lambs.

## 2. Material and Methods

### 2.1. Location in Which Experiment Was Performed and Ethical Considerations

This experiment was conducted at the Universidad Autónoma of Sinaloa Feedlot Lamb Research Unit, located in Culiacán City (24°46′13″ N, 107°21′14″ W; 55 m a.s.l.), México. Culiacán City has a tropical climate. The experiment was carried out from March to May 2023, and the average climatic conditions that occurred during the experimental period were 29.1 ± 2.8 °C ambient temperature and 33.3 ± 7.3% relative humidity (RH). All animal management procedures were conducted within the guidelines of federally and locally approved techniques for animal use and care [12] and approved by the Ethics Committee of the Faculty of Veterinary Medicine and Zootechnics of the Autonomous University of Sinaloa (Protocol #06132023).

### 2.2. Animals, Diet, and Experimental Design

Thirty-six Pelibuey× Katahdin male intact lambs (158 ± 22 days of age, 18.81 ± 3.04 kg initial live weight) were used to evaluate the effects of feeding a high-energy diet supplemented with AZOMITE, a source of hydrated sodium calcium aluminosilicate (AZO), on growth performance, dietary energy utilization, and carcass characteristics of finishing lambs. Four weeks before the initiation of the experiment, all lambs were treated for parasites (Albendaphorte 10%, Animal Health and Welfare, México City, México), injected with 1×10^6^ IU vitamin A (Synt-ADE^®^, Fort Dodge, Animal Health, México City, México), and vaccinated for Mannheimia haemolytica (One Shot; Zoetis, México City, Mexico). All lambs were adapted to the finishing diet (without AZO supplementation, Table 1) and facilities 21 days before the start of the experiment. The basal diet was prepared using a 2.5 m^3^ capacity paddle mixer (model 30910-7, Coyoacán, México). To avoid contamination, the mixer was thoroughly cleaned before the start of each dietary treatment. To determine nutrient composition, a feed sample (~50 g) from each batch was taken and stored (4 °C) in sealed bags. Upon initiation of the experiment, all lambs (*n* = 36) were weighed before the morning meal (electronic scale ±0.05 kg precision; TORREY TIL/S: 107 2691, TORREY Electronics Inc., Houston TX, USA), and assigned within six weight groupings to 18 pens, with two lambs per pen and 6 replicates per treatment. Pens were 6 m^2^ with overhead shade, plastic bucket waterers and 1 m fence-line feed bunks. The pens’ floor was composed of a compacted earth floor covered with special soft absorbent loamy/sandy soil technically named “tucuruguay”. Due to its softness and absorbance, no bedding material was necessary. Although there is no scientific information regarding optimal levels of AZO inclusion in ruminant diets, the manufacturer indicated that, in accordance with good feed manufacturing or feeding practices, it is recommended to add a maximum of 2% of AZOMITE^®^ to the diet. Therefore, treatments consisted of a total mixed-ration corn-based diet with 88:12 concentrate-to-forage (Sudangrass hay) ratio supplemented (based on dry matter, DM) as follows: (1) no AZO inclusion (CTRL), (2) AZO inclusion at 0.75% level (0.75AZO) in diet DM, and (3) AZO inclusion at 1.50% level (1.5AZO) in diet DM. Cracked corn was replaced in the control diet by AZO according to the treatment. The experiment lasted 81 days. The source of the hydrated sodium calcium aluminosilicate used was AZOMITE (AZOMITE Mineral Products Inc., Nephi, UT, USA), the mineral composition of which is shown in Table 2. The respective amount of AZO for each treatment was premixed with mineral–protein supplement before incorporation into complete mixed diets, which were formulated to meet or exceed nutrient requirements for daily weight gain of 250 g (Table 1) [13]. Fresh feed was provided twice daily at 0800 and 1400 h in a 40:60 proportion of the total daily feed consumption (as feed basis). Whereas the amount of feed provided in the morning feeding was constant, feed offered in the afternoon feeding was adjusted daily, allowing for a residual ~50 g/kg daily feed offering. Residual feed of each pen was collected between 0740 and 0750 h each morning, was composited through the experiment and weighed on the final day of the experiment to measure the feed intake. The adjustments to either increase or decrease daily feed delivery were provided in the afternoon feeding. Water consumption was measured daily at 0700 h by dipping a graduated rod into the plastic bucket drinker (one watering tank for each pen collocated at 40 cm height and fixed to the pen). Once the measure was taken, water intake was estimated by difference and the remaining water was drained, and the tanks were refilled with fresh water. Lambs were individually weighed prior to the morning feeding (0730 h) at experiment initiation and on days 28, 56, and on final day (day 81, harvest). Live weights (LW) on day 1 were converted to shrunk body weight (SBW) by multiplying LW by 0.96 to adjust for the gastrointestinal fill. All lambs were fasted (for feed but not for drinking water) for 18 h before recording the final fasted LW prior to harvest. 

### 2.3. Feed and Refusals Analysis 

Feed samples were collected for each elaborated batch. Feed refusal was collected daily and composited weekly for dry matter analysis (DM; oven drying at 105 °C until no further weight loss, method 930.15) [14]. Feed samples were subjected to the following analyses: DM (oven drying at 105 °C until no further weight loss; method 930.15) and CP (N× 6.25, method 984.13) according to AOAC [14]. Neutral detergent fiber (NDF) was determined following procedures described by Van Soest et al. [15] (corrected for NDF ash, incorporating heat-stable α-amylase using Ankom Technology, Macedon, NY, USA).

### 2.4. Calculations

Estimates of average daily weight gain (ADG) and dietary net energy (NE) are based on initial SBW and final (d 81) fasted SBW. The average daily gain was computed by subtracting the initial SBW from final SBW and dividing the result by the number of days on feed. Average dry matter intake (DMI) was calculated by dividing total intake during the experiment by 81 (total days on feed). Feed efficiency was computed as ADG/average DMI observed during the 81 days of the experiment. One approach for evaluation of the efficiency of dietary energy utilization in growth performance trials is the ratio of observed-to-expected DMI and observed-to-expected dietary NE. Based on estimated diet NE concentration and measures of growth performance, there is an expected energy intake. This estimation of expected DMI is performed, as follows, based on observed ADG, average SBW, and NE values of the diet (Table 1): expected DMI, kg/d = (EM/EN_m_ diet) + (EG/EN_g_ diet), where EM (energy required for maintenance, Mcal/d) = 0.056 × SBW^0.75^, EG (energy required for gain, Mcal/d) = 0.276 × ADG × SBW^0.75^; and the NE_m_ and NE_g_ were the values contained in the diet of each treatment (Table 1). Those values were calculated based on the ingredient composition in the basal diet (Table 1). The coefficient (0.276) was taken from NRC [16], assuming a mature weight of 113 kg for Pelibuey × Katahdin male lambs. The observed dietary net energy was calculated using EM and EG values and the DMI observed during the experiment by means of the quadratic formula:

(1) $$x=\frac{-b\pm \sqrt{b^{2}-4ac}}{2c}$$

where *x* = observed dietary NE_m_, Mcal/kg; *a* = −0.41 EM; *b* = 0.877 EM + 0.41 DMI + EG; and *c* = −0.877 DMI [17].

### 2.5. Carcass Dressing and Visceral Organ Mass

All of the lambs were harvested on the same day. After humanitarian sacrifice, lambs were skinned, and the gastrointestinal organs were separated, and hot carcass weight (HCW) was recorded. Dressing percentage (DP) was calculated by dividing HCW/final shrunk weight. All tissue weights were reported on a fresh-tissue basis. Organ mass was expressed as grams of fresh tissue per kilogram of final empty body weight (EBW). Final EBW represents the final LW minus the total digesta weight. The stomach complex was calculated as the digesta-free sum of the weights of the rumen, reticulum, omasum, and abomasum.

### 2.6. Statistical Analysis

All of the data were tested for normality using the Shapiro–Wilk test. Growth performance data (gain, gain efficiency, and dietary energetics), DM intake, and carcass data were analyzed as a randomized complete block design with the pen as the experimental unit, using the MIXED procedures of SAS software (version 9.3) [18], with treatment and block as fixed effects and the experimental unit within treatment as a random effect. Water intake was analyzed as a completely randomized design using linear mixed model for analysis of repeated measures with the pen as the experimental unit [18]. In all cases, contrasts are considered significant when the *p* value < 0.05, and tendencies were identified when 0.05 < *p* ≤ 0.10.

## 3. Results

No morbidity or mortality was observed during the experimental period, and no lamb was retired from the experiment. The average climatic conditions that occurred during the experimental period were 29.1 ± 2.8 °C ambient temperature and 33.3 ± 7.3% relative humidity (RH). Those values of ambient temperature and RH represent a temperature humidity index (THI) of 74.7; thus, lambs were finished under favorable environmental conditions [19]. The treatment effects on performance and carcass dressing percentage are shown in Table 3. Because water consumption, feed consumption, and daily gain showed the same trend between treatments during periods 1–28, 29–56, and 57–81, only data from the global period (1–81 days) are reported. Water intake was not affected (*p* ≥ 0.39) by treatments, averaging 3.1 L/d. Daily ingestion of AZO averaged 7.15 and 15.3 g/lamb. Dry matter intake was not affected (*p* ≥ 0.11) by AZO supplementation, but as a result of a tendency (*p* = 0.06) to increase ADG in lambs supplemented with AZO, the inclusion of AZO in the diet increased gain efficiency and observed dietary energy, being maximal with 0.75% AZOMITE inclusion (quadratic effect, *p* = 0.02). The observed-to-expected dietary NE in the control group was as anticipated (1.00) according to the estimated energy based in the ingredient composition in the diet (Table 1), while in the 0.75AZO group, the observed dietary energy was 6% above the expected (1.06), indicating a greater efficiency in energy utilization destined to growth (quadratic effect, *p* < 0.006). Lambs that were fed the AZO treatment showed a linear increase (*p* = 0.04) in hot carcass weight (HCW), and tended (*p* = 0.06) to linearly increase dressing percentage (DP). Visceral organ mass (expressed as g/kg of empty body weight) was not affected by the AZO inclusion in the diet (Table 4).

## 4. Discussion

Currently, the livestock production system is undergoing a slow but progressive conversion to the use of safe feed additives and/or functional feeds. The concern on the part of consumers to access products free of compounds such as antibiotics has led governments to establish increasingly strict rules for the regulation of these and other compounds that impact the safety of the final product. This has led to the search for alternatives that can replace these compounds but obtain the same results in productivity and health [20,21]. Among these, the use of volcanic clays as a feed additive in animal diets has been gaining greater interest in recent years [1]. Contrary to AZO, volcanic clays such as zeolite and bentonite have been extensively studied as feed additives for ruminant diets. Clays in general are characterized by their high cation exchange capacity and high specific surface area [22,23]. In addition, they disperse easily in an aqueous medium, modifying their viscosity [24]. Because the clays have a binding capacity with certain compounds which have nutritional and health importance, clays are used in the agriculture and livestock industry. Although clays have a certain similarity in mineral composition, depending on the region where they are formed, there are particularities regarding the presence and/or concentration of certain minerals that provide them with special physical and chemical characteristics [3]. This is mainly in regard to the presence and concentration of rare earth elements in clays. As is mentioned above, there is no information available about the effect of AZO supplementation on the growth performance and carcass characteristics of ruminants. Because information about AZO in ruminants is not available, some of our results compared it, with the proper proportion of the case, with zeolite, the main clay investigated as a feed additive for ruminants. The inclusion of zeolite from 2 to 4% in ruminant diets has been shown to promote changes in ruminal fermentation and nutrient digestion that promotes improvements in feed efficiency [25,26,27,28,29,30].

There is no evidence of the effects on the water intake of ruminants when they have been fed clays as feed additives. It has been argued that the effect of clays on digestion can be attributed to the influence on rumen retention time or the alteration of the turnover of fluid and particulate phases of digesta. These effects could affect the water intake behavior; however, Sweeney et al. [25] noted no differences in fluid digesta flow from the rumen of steers supplemented with 3% zeolite. Based on our results, it can be expected that AZO supplementation, included at the levels used here, does not affect the water intake of lambs fed high-energy diets.

In line with our results, DM intake commonly is not affected by zeolite supplementation from 2 to 6% in finishing diets for ruminants [26,27,28,29,30]. Similarly, HSCAS supplementation up to 4% did not affect DM intake in dairy goats [31], and particularly, AZO supplementation did not affect DM intake in broilers [32], laying hens [33], pigs [34], and fishes [35].

The improvements in performance and feed efficiency in broilers and pigs by zeolite supplementation have been extensively reported [1,21]. In ruminants that have been fed high-grain diets, it has been reported that zeolite supplementation enhanced ruminal starch digestion and changed ruminal molar proportions of VFAs, decreasing acetate-to-propionate ratios [36,37]. Zeolite supplementation helps ruminants to maintain rumen pH, improving organic matter degradability and total VFA concentration and reducing the ruminal concentration of NH_3_-N and CH_4_ [28,38]. These effects may decrease methanogenesis and increase the ruminal fermentation efficiency. However, despite the potential beneficial effects on ruminal fermentation and nutrient digestion, supplemental zeolite has not consistently improved animal performance. In a trial performed by Forouzani et al. [39] using Mehraban lambs, a 3% inclusion of zeolite in the diet, increased the total tract digestion of DM, CP, and NDF and decreased ruminal pH and blood urea nitrogen, but the growth performance and feed efficiency was not different to the control group lambs. In the reports which state that zeolite supplementation has positive effects on ruminants’ performance, it either improves daily gain or feed efficiency, but not both [40,41]. Roque-Jiménez et al. [28] noted an increase in ADG, but not in feed efficiency when Rambouillet ewe lambs were fed a moderated-energy diet containing 4% supplemental zeolite for 52 d. Therefore, the increase in daily gain was directly attributed to the increase in DM intake (and energy) of lambs supplemented with zeolite. Meanwhile, Estrada-Angulo et al. [27] observed an improvement in feed efficiency, but not in ADG, when Pelibuey x Katahdin lambs were supplemented with 3% zeolite in a partial replacement of corn and soybean meal in a high-energy finishing diet. However, most reports indicate that clay supplementation in diets for lambs does not affect the feed efficiency [42,43]. In this context, Estrada-Angulo et al. [26] did not detect improvements in feed efficiency when zeolite was included up to 3% in a partial replacement of soybean meal in high-energy finishing diets offered to hairy lambs for 56 d. Similarly, the absence of an effect of clay supplementation on feed efficiency in Santa Ines lambs that were fed up to 7% zeolite in a 40:60 concentrate-to-forage ratio for 91 d has been reported by Esteves et al. [41]. In the same line, Merino × Ile de France crossbred lambs that were fed a moderated-energy diet (1.93 Mcal/kg of NE_m_) showed no differences in feed efficiency when supplemented with 2% zeolite [43].

Because of the great controversy of the results observed with clays as feed additives on feed efficiency, it has been argued that the magnitude of the response to clay supplementation can be mediated by the supplementation level, mineral status of the animals and mineral concentration in diet, type of diet (i.e., high-concentrate diets), and the presence of contaminants and pathogenic agents (certain parasites and bacteria) in the diet that affect productivity and health, among others [1,8,21]. However, the majority of studies have not considered that clays, which are included as a functional ingredient in diets, having no intrinsic energy value; furthermore, their substitution in the diet as a replacement for feed ingredients will reduce the energy density of the diet in proportion to the replacement. And this point was not considered when the feed efficiency analysis was performed. This bias of comparison between treatments with different energy concentrations can be adjusted by using the observed-to-expected dietary net energy ratio instead of the gain-to-feed ratio.

The relationship between diet energy density based on tabular values for individual feed ingredients and estimated diet energy density based on growth performance is an effective method for assessing the efficiency of dietary energy utilization [17,44]. A ratio of observed/expected dietary NE of 1.00 indicates that observed ADG is consistent with the expected ADG based on DM intake and formulated dietary energy density. A ratio of less than 1.00 indicates a lower-than-expected energetic efficiency, and vice versa for a ratio greater than 1.00. This energetic approach to evaluate the impact of supplemental clays on dietary energy efficiency in ruminants has been clearly exposed by Urías-Estrada et al. [44]. Under this analysis, the data of several reports indicate that supplemental clays improve observed dietary energy by around 3.5% when they are supplemented at levels of 2 to 3% in diets for lambs [26,27,29,30,39,41,43]. In the present experiment, compared to controls, AZO supplementation at 0.75% tended (*p* = 0.06) to increase ADG by 4.6%, increased gain efficiency by 5.5% (*p* = 0.02), and observed dietary energy by 4.2%(*p* < 0.01); the increase in observed dietary energy is equivalent to a 6% increase in dietary energy utilization (observed-to-expected diet NE). Thus, in order to improve the energy efficiency of a diet when supplemented by AZO, it should be supplemented at a quarter of the dosage that is needed for zeolite to do so. The current study provides new insights into the use of AZO in finishing diets for lambs.

The basis of the apparently greater potency of AZO than zeolite related to dietary energy efficiency is uncertain and required more research. One of the possible explanations is that AZO contains up to five times more mineral elements than other members of the aluminosilicate family (i.e., zeolites) commonly used in animal feeds [4]. The greater content in the mineral profile for AZO could represent an advantage for animal productivity. It is well known that supra-supplementation of certain trace minerals (i.e., chromium, Zn, Cu) can favor physiological changes that promote improvements in energy utilization from the feed via changes in the composition of gain or via a reduction in energy requirements for maintenance [45,46,47]. On the other hand, AZO is rich in rare earth elements (REEs), mainly lanthanides. The mechanism of REEs for enhancing animal performance is not well understood and the scientific literature on it is limited. However, different biological effects can be attributed to REEs in animals based on the results of different feeding trials. Aside from the antibacterial and antioxidant properties of REEs, they also stimulate hormone production, enzyme activity, and immune system functions, and, finally, they improves digestibility and nutrient utilization [48,49]. In growing bulls, REE supplementation improves feed efficiency [48]. Lanthanide supplementation improves ADG and feed efficiency when fed (100–300 mg/kg diet) to African dwarf sheep, while in Simmental steers, lanthanide supplementation improves rumen fermentation and nutrient digestion via stimulation of ruminal microorganisms and enzyme activity [50]. In contrast, Schwabe et al. [51] reported decreases in weight gain and an absence of effects on nutrient digestion when Holstein bulls were supplemented with REE citrate from 100 to 300 mg/kg diet. Based on certificate analysis, AZO contained around 469 mg REE/kg AZO (Table 2); thus, the estimated daily ingestion of REEs averaged 5.3 mg, which is far away from the doses used in previous reports that evaluated REE effects as feed additives.

Regardless, the minimal information regarding AZO supplementation indicates, in consistency with our results, the positive effects of AZO as a feed additive on improvements in ADG or feed efficiency in non-ruminant and fish species. In this sense, inclusions of 0.25 to 1.0% of AZO in diets for broilers have improved weight gain and feed efficiency [5]. Increases in the growth rate of 5.6% and 7.8% in feed efficiency in broilers were explained by increases in nutrient digestibility and by improvements in mineral retention [7]. In a study performed by Juzaitis-Bolter et al. [33], they observed that AZO supplementation at level of 0.25% of the diet increased growth performance and feed efficiency in broilers during the first 21 d of age, and egg production and feed efficiency in laying hens from 54 through 98 weeks of age. Similar positive responses in growth performance, feed efficiency, and health had been obtained when diets for fishes were supplemented by AZO [9,52]. However, some reports indicate that AZO supplementation did not show any advantages for ADG or feed efficiency when it was supplemented at level of 0.5% in diets for pigs [34] and broilers [8].

There was no information about the impact of supplemental ZEO in the carcass of ruminants. The increase in HCW observed here can be explained by the greater daily weight observed in lambs that were fed ZEO. It is well known that one of the main factors that affect HCW is the rate of weight gain [53,54]. Reports on the effect of supplementing ruminant diets with clays on the carcass dressing percentage have been inconsistent. Some reports indicate that the carcass dressing percentage was not affected by zeolite supplementation up to 3% [26,29,55], whereas in others, carcass dressing was reduced in Holstein steers by zeolite supplementation when its inclusion in diets ranged from 3 to 5% [56,57]. In a research work performed by Coronel-Burgos et al. [55], in which hairy lambs were fed a high-energy diet supplemented by up to 4% zeolite, they noted that zeolite changed the composition of gain by more muscle and less fat deposited in the carcass; these changes could favor a greater dressing percentage in the carcass. However, more research is still needed to know more precisely the effects of ZEO supplementation on the carcass characteristics of ruminants. There were no previous reports regarding AZO’s effects on ruminants’ visceral mass. However, the inclusion of clays in finishing diets up to 1.5% did not affect the visceral mass of lambs [26,55].

Although the purpose of this study was not to perform an economic assessment, it is important to note that, due to the AZO inclusion improving feed efficiency and increased hot carcass weight, lambs that received AZO resulted in more income than non-supplemented lambs. The price of AZO is $1.00 US dollar/kg; thus, the cost of inclusion during 81 d of fattening were $0.58 and $1.16 US dollars for 0.75 and 1.5% of AZO in diet, respectively. The extra income by differential for carcass weight/lamb were $4.70 and $7.05 US dollars. In this manner, the net incomes/lamb were 4.20 and 5.89 US dollars for lambs that received 0.75 and 1.5% of AZO in their diet, respectively.

## 5. Conclusions

Under the conditions in which this experiment was conducted**,** it was concluded that up to 1.5% AZOMITE clay can be included in finishing diets for lambs without detrimental effect on the intake of water and feed. Lambs that received the diet that contained 0.75% showed greater efficiency in retaining dietary energy, improving the feed-to-gain ratio. Due to the slight energy dilution caused by the AZO inclusion in their diet, the daily energy intake of lambs which received 0.75% AZO was 2.5% lower, but ADG tended to be greater than the control lambs; therefore, the observed dietary energy was 6% above the expected, indicating a greater efficiency in energy utilization destined to growth. Increasing AZO supplementation beyond 0.75% in the diet did not improve energy efficiency but improved HCW and tended to increase the dressing percentage of the carcass. This is the first report regarding the effects of AZO supplementation in the dietary energy utilization of fattening hairy lambs. Further research about the effects of AZO supplementation on the ruminal and total tract digestion, carcass and meat quality, and health of lambs is needed in order to more deeply understand the effects of AZO on productivity performance.

## Figures and Tables

**Table 1:** Composition of experimental diets (total mixed diets) fed to lambs (DM basis).

Item	AZOMITE Inclusion in Diet (%)		
	0.00	0.75	1.50
Ingredient Composition (%, DM)			
Cracked corn grain	60.00	59.25	58.50
AZOMITE ^1^	0.00	0.75	1.50
Soybean meal	14.00	14.00	14.00
Sudan grass hay	12.00	12.00	12.00
Molasses cane	8.00	8.00	8.00
Yellow grease	3.50	3.50	3.50
Mineral–protein supplement ^2^	2.50	2.50	2.50
Dry matter content (%)	88.23	88.88	88.93
Nutrient Composition (DM basis) ^3^			
Net energy (Mcal/kg)			
Maintenance	2.13	2.11	2.09
Gain	1.46	1.44	1.43
Crude protein (%)	14.73	14.62	14.58
NDF (%)	16.52	16.40	16.32
Ether extract (%)	6.30	6.28	6.28
Mineral matter	6.15	6.89	7.62

^1^ Volcanic ash composed by hydrated sodium calcium aluminosilicate distributed by AZOMITE Mineral Products Inc., Utah, USA. ^2^ Corn grain was prepared and adjusted so that the kernels were broken to produce a bulk density of approximately 0.60 kg/L. Mineral–protein supplement contained the following (%): crude protein 72.8%, calcium, 20%; CoSO4, 0.010%; CuSO4, 0.15%; FeSO4, 0.528%; ZnO, 0.111%; MnSO4, 0.160%; KI, 0.007%; and NaCl, 13.7%.^3^ Sudangrass hay was ground in a hammer mill (Azteca 20, Molinos Azteca, Guadalajara,México) with a 3.81 cm screen before incorporation into a total mixed ration. Calculated based on tabular values for individual feed ingredients [13] with the exception of CP and NDF, which were determined in our laboratory.

**Table 2:** Main minerals and total rare earth elements contained in AZOMITE.

Mineral	% Unless Shown as mg/kg
Alumina	6.57%
Calcium oxide	3.67%
Silicon dioxide	65.85%
Potassium oxide	5.23%
Ferric oxide	1.37%
Sodium oxide	2.07%
Total rare earth elements (mg/kg)	479

Data adapted from AZOMITE ore certificate of analysis.

**Table 3:** Influence of AZOMITE inclusion on growth performance and dietary energy of feedlot lambs.

Item	AZOMITE in Diet (% of Diet DM)				Contrast *p* Value	
	0.00	0.75	1.50	SEM	L	Q
Water intake (L/d)	3.15	2.99	3.13	0.138	0.90	0.39
Live Weight (kg) ^1^						
Initial	18.92	18.88	18.75	0.09	0.21	0.75
Final	40.19	41.18	41.44	0.39	0.09	0.56
Daily gain (kg)	0.263	0.275	0.280	0.006	0.06	0.17
Dry matter intake (kg/d)	0.965	0.954	1.017	0.020	0.11	0.26
Gain to feed (kg/kg)	0.273	0.288	0.275	0.003	0.62	0.02
Observed Dietary Net Energy (Mcal/kg)						
Maintenance	2.13	2.22	2.14	0.019	0.84	0.008
Gain	1.46	1.53	1.46	0.016	0.84	0.007
Observed-to-Expected Dietary NE						
Maintenance	1.00	1.06	1.02	0.009	0.18	0.007
Gain	1.01	1.07	1.02	0.011	0.23	0.006
Hot carcass weight (kg)	23.85	24.79	25.26	0.421	0.04	0.66
Dressing percentage	59.20	60.15	60.87	0.564	0.06	0.87

^1^ The initial BW was reduced by 4% to adjust for the gastrointestinal fill, and all lambs were fasted (food but not drinking water was withdrawn) for 18 h before recording the final BW.

**Table 4:** Influence of AZOMITE inclusion on visceral organ mass of feedlot lambs.

Item	AZOMITE in Diet (% of Diet DM)				Contrast *p* Value	
	0.00	0.75	1.50	SEM	L	Q
Full viscera (kg)	8.218	8.477	8.122	0.190	0.73	0.22
Organs (g/ kg of Empty Body Weight)						
Stomach complex	25.33	25.66	24.83	1.164	0.76	0.69
Intestines	33.50	32.83	32.50	1.253	0.58	0.91
Heart lungs	19.33	20.16	19.00	0.920	0.80	0.39
Liver + spleen	18.66	18.83	18.00	0.802	0.57	0.62
Kidney	2.67	3.00	2.33	0.235	0.34	0.12

## Data Availability

The data that support the findings of this study are available from the corresponding author upon reasonable request.
